# Recognition Functions of Pentameric C-Reactive Protein in Cardiovascular Disease

**DOI:** 10.1155/2014/319215

**Published:** 2014-05-19

**Authors:** Alok Agrawal, Toh B. Gang, Antonio E. Rusiñol

**Affiliations:** Department of Biomedical Sciences, Quillen College of Medicine, East Tennessee State University, Johnson City, TN 37614, USA

## Abstract

C-reactive protein (CRP) performs two recognition functions that are relevant to cardiovascular disease. First, in its native pentameric conformation, CRP recognizes molecules and cells with exposed phosphocholine (PCh) groups, such as microbial pathogens and damaged cells. PCh-containing ligand-bound CRP activates the complement system to destroy the ligand. Thus, the PCh-binding function of CRP is defensive if it occurs on foreign pathogens because it results in the killing of the pathogen via complement activation. On the other hand, the PCh-binding function of CRP is detrimental if it occurs on injured host cells because it causes more damage to the tissue via complement activation; this is how CRP worsens acute myocardial infarction and ischemia/reperfusion injury. Second, in its nonnative pentameric conformation, CRP also recognizes atherogenic low-density lipoprotein (LDL). Recent data suggest that the LDL-binding function of CRP is beneficial because it prevents formation of macrophage foam cells, attenuates inflammatory effects of LDL, inhibits LDL oxidation, and reduces proatherogenic effects of macrophages, raising the possibility that nonnative CRP may show atheroprotective effects in experimental animals. In conclusion, temporarily inhibiting the PCh-binding function of CRP along with facilitating localized presence of nonnative pentameric CRP could be a promising approach to treat atherosclerosis and myocardial infarction. There is no need to stop the biosynthesis of CRP.

## 1. Introduction

C-reactive protein (CRP) is a multifunctional and evolutionarily conserved plasma protein (reviewed in [1–8]). Through the circulation, CRP reaches tissues and is seen deposited at sites of inflammation. Human CRP is comprised of five identical subunits arranged in a cyclic pentamer [9]. In this paper, we review two recognition functions of pentameric CRP which are relevant to cardiovascular disease: the phosphocholine- (PCh-) binding function of native pentameric CRP that has been implicated in acute myocardial infarction and ischemia/reperfusion (I/R) injury and the atherogenic low-density lipoprotein- (LDL-) binding function of nonnative pentameric CRP that has been implicated in atherosclerosis.

## 2. PCh-Binding Function of Native Pentameric CRP, Myocardial Infarction, and I/R Injury

A major function of CRP in its native pentameric form is to bind, in a Ca^2+^-dependent manner, to molecules and cells bearing exposed PCh groups, such as the cell wall of pneumococci and cell membrane of damaged cells [10, 11]. Once CRP is bound to a PCh-containing ligand, it activates the complement system to destroy the ligand [12, 13]. When CRP binds to foreign pathogens, it helps in the killing of the pathogen via complement activation. In mouse models of pneumococcal infection, CRP has been shown to be protective; that is, CRP decreases bacteremia and increases survival of infected mice ([14] reviewed in [15, 16]). Experiments performed* in vitro* using necrotic and apoptotic cells reveal that the binding of CRP to necrotic and apoptotic cells can facilitate the removal of such cells [17–21]. However, experiments performed* in vivo* using animal models of I/R injury reveal that the binding of CRP to damaged cells is detrimental to the tissue [22–25]. Combined data suggest that the consequences of the binding of CRP to damaged cells depend on the tissue. In many places in the body (skin and subcutaneous tissue, e.g.,), it does no harm to bind complement and hasten death of dead tissue. The situation for the organs which are working all the time and do not have the ability to regenerate their tissue (heart, e.g.,) is different and hastening removal of dead tissue will be harmful. During myocardial infarction, the necrotic part of the myocardium will be removed by CRP. However, the ischemic part of the tissue where the damage can be reversed may also be removed by CRP, as described previously [26]. Thus, the PCh-binding function of CRP is defensive for the host because it leads to protection against pneumococcal infection and removal of necrotic tissue. On the other hand, the PCh-binding function of CRP is detrimental for the host when CRP binds to reversibly damaged myocardial cells, because it causes more damage to the tissue via complement activation.

Studies in animals (mice, rats, and rabbits) and human specimens have shown that both CRP and components of the activated complement system are deposited and colocalized in myocardial infarcts and that complement activation is due to the presence of CRP [27–32]. CRP has been shown to exacerbate left ventricular dysfunction and promote adverse left ventricular remodeling after myocardial infarction [33]. Mostly by employing animal models of I/R injury, it has been shown that CRP enhances the size of myocardial infarcts and also contributes to ischemic tissue damage in intestine, lung, kidney, and brain [22–25, 32–34]. In a mesenteric I/R model, CRP deposition correlated with complement deposition, suggesting a role of CRP in complement activation [23]; in these studies, inhibition of complement activation by using C1 inhibitor reduced the effects of CRP on intestinal injury. Similarly, inhibition of complement activation by decay-accelerating factor also prevented CRP-mediated intestinal injury and remote lung damages following mesenteric I/R [24]. In mice transgenic for human CRP, arterial injury resulted in an expedited and higher rate of thrombotic occlusion compared to that in nontransgenic mice [35]. CRP-mediated exacerbation of vascular injury involves complement since lowering the biosynthesis of CRP prevented complement consumption [36]. These findings indicated that an intact complement system is required for the damaging effects of CRP on myocardial injury because lowering of CRP level, depleting complement, or blocking CRP-mediated complement activation abrogated the effects of CRP. Thus, CRP- and CRP-mediated complement activation both contribute to myocardial injury.

Each subunit of CRP has a PCh-binding site. The three-dimensional structure of the PCh-binding site reveals that Glu81 in the PCh-binding hydrophobic pocket of CRP interacts with the nitrogen atom of choline in PCh, that Phe66 interacts with the three methyl groups of choline, and that Thr76 is critical for creating the appropriately sized pocket on CRP to accommodate PCh. The phosphate group of PCh directly coordinates with the two calcium ions bound to CRP [9, 37]. We generated a CRP triple mutant, F66A/T76Y/E81A, that does not bind to PCh and was therefore unable to form complexes capable of activating complement [14]. Such a mutant is suitable for use in experiments aimed at defining the contribution of the PCh-binding site of CRP in deteriorating tissue injury. In another approach, pharmacological inhibition of CRP using a PCh-based compound reduced the deposition of CRP at myocardial infarcts and inhibited complement activation, indicating that the PCh-binding site of CRP participates in worsening the infarct size and that the inhibition of the PCh-binding site is a useful strategy to prevent tissue-damaging conditions [38]. Similarly, pharmacological inhibition of biosynthesis of CRP also resulted in a reduction of CRP-mediated exacerbation of vascular injury [36].

## 3. LDL-Binding Function of Nonnative Pentameric CRP and Atherosclerosis

Native CRP does not bind to native LDL under normal physiological conditions [39–42]. Native CRP and native LDL interact with each other only when either one is immobilized, modified, or aggregated [39–44], raising the possibility that CRP and LDL may interact with each other under pathological conditions. The native pentameric structure of CRP can be modified* in vitro* and we have shown that the recognition functions of nonnative pentameric CRP are different from those of native CRP: one function of CRP in its nonnative pentameric conformation is to bind to atherogenic LDL [45–49]. Two types of LDL are used as models of atherogenic LDL: enzymatically modified LDL (E-LDL) and oxidized LDL (ox-LDL) [50–53]. To E-LDL, even native CRP binds and the binding is inhibited by free PCh [40, 45, 54]. Data obtained from PCh-inhibition experiments suggest that CRP binds to E-LDL through the PCh groups in E-LDL and that the binding is mediated by the PCh-binding site of CRP. However, the amino acids in CRP that contact PCh are not critical for the binding of CRP to E-LDL, indicating that the PCh groups present in E-LDL are not the only components in E-LDL through which CRP binds to E-LDL [45]. It has been shown that CRP binds to E-LDL through cholesterol also and that this binding was also PCh-inhibitable [55, 56]. Nonnative CRP binds to E-LDL more avidly than native CRP through an as-yet-undefined mechanism [45–47].

Several investigators have reported that native CRP can also bind to ox-LDL through the PCh moiety in ox-LDL [41, 54, 57] and several investigators have reported that native CRP does not bind to ox-LDL [42, 47, 48, 55, 58]. CRP has also been shown to bind to ox-LDL* in vivo* in diabetes mellitus patients with atherosclerosis and when ox-LDL is complexed with *β*2 glycoprotein I [59, 60]. We reported that a modification of the native pentameric structure of CRP was required for binding to ox-LDL and that CRP, in its nonnative pentameric conformation, binds efficiently to ox-LDL [46–48]. Taken together, it seems that the binding of CRP to ox-LDL depends on the stringency of the method used to prepare ox-LDL. If the PCh groups are exposed to ox-LDL, then native CRP would bind, if the PCh groups are not exposed to ox-LDL, then native CRP would not bind, and nonnative CRP would bind to ox-LDL regardless of the extent and nature of oxidation. The mechanism of interaction between nonnative CRP and atherogenic LDL, however, has not been elucidated yet. On the LDL molecules, the moieties that could interact with CRP include PCh, cholesterol, apoB, and phosphoethanolamine [40, 41, 43, 45, 51, 52, 55, 56, 61, 62]. In addition, the amyloid-like structures which are induced in LDL by oxidation could also be recognized by nonnative CRP [63]. We hypothesize that the LDL-binding site is buried (or absent) in native CRP and is exposed (or formed) in nonnative CRP by the loosening up of the pentamer [47, 48]. CRP has been found deposited and colocalized with LDL in atherosclerotic lesions in humans and experimental animals, indicating the presence of nonnative CRP at the lesions [64–70].

The recognition function of CRP to bind to atherogenic LDL should have an effect on the formation of LDL-loaded macrophage foam cells and also on the proinflammatory effects of foam cells and LDL. The formation of foam cells represents an early step in atherosclerosis and begins when macrophages bind and take up LDL [71–73]. Using immunohistochemical staining of atherosclerotic lesions with antibodies to CRP and LDL, the outcome of the interactions among native or aggregated CRP, LDL, and macrophages with regard to the formation of macrophage foam cells has been investigated extensively [44, 57, 65, 74–76]; however, a review of the published literature does not provide a clear-cut overall conclusion [1, 77]. Similarly, it is also unclear whether both Fc*γ* receptor CD32 and LDL receptor CD36 on macrophages participate if there is an effect of CRP on the uptake of LDL by macrophages [44, 57, 76, 78]. We investigated the effect of CRP on the accumulation of lipid droplets made up of cholesteryl esters in E-LDL-treated macrophages and found that, in contrast to E-LDL alone, CRP-bound E-LDL was inactive for the formation of foam cells [45]. Other consequences of CRP-LDL interactions have also been reported [74, 79–81]. CRP causes charge modification of LDL [74]. CRP reduces the susceptibility of copper-induced oxidation of LDL [58, 79]. CRP attenuates adhesion and activation of monocytes via the prevention of binding of minimally modified LDL to monocytes; this effect was mediated by the binding of CRP to monocytes [80]. CRP also suppresses the proatherogenic effects of macrophages when bound to lysophosphatidylcholine, a moiety present in oxLDL [81]. Collectively, these findings suggest that CRP, under defined conditions, prevents foam cell formation and reduces proinflammatory effects of LDL and foam cells.

To determine the role of CRP in the development of atherosclerosis, human native CRP has been introduced into three different murine models of atherosclerosis:* ApoE* ^−/−^ mice,* LDLr* ^−/−^ mice, and* ApoB* ^100/100^ *Ldlr* ^−/−^ mice (reviewed in [1, 77]). CRP was found to be neither proatherogenic nor atheroprotective in* ApoE* ^−/−^ mice [82–85]. Both passively administered CRP and transgenically expressed CRP had no effect on the development, progression, or severity of spontaneous atherosclerosis in* ApoE* ^−/−^mice. In* LDLr* ^−/−^mice also, there was no effect of CRP on the development of atherosclerosis [86]. In rabbits transgenic for human CRP also, CRP did not affect aortic or coronary atherosclerosis lesion formation [87]. However, two recent studies indicated atheroprotective effects of CRP [88, 89]. In* ApoB* ^100/100^ *Ldlr* ^−/−^ mice, CRP slowed the development of atherosclerosis [88]. In* ApoE* ^−/−^ *CRP* ^−/−^ and* LDLr* ^−/−^ *CRP* ^−/−^ mice, the size of atherosclerotic lesions was either equivalent or increased when compared to that of* ApoE* ^−/−^ and* LDLr* ^−/−^ mice, suggesting that even mouse CRP may mediate atheroprotective effects. These data raise hopes that nonnative CRP may be more atheroprotective than native CRP considering the difference between the LDL-binding recognition functions of nonnative and native CRP.

Although much more experimentation needs to be done, there are already several lines of evidence to indicate that the LDL-binding function of CRP is beneficial and may contribute to atheroprotection. First, CRP reduces further oxidation of LDL. Second, CRP attenuates monocyte adhesion and activation via the prevention of binding of atherogenic LDL to monocytes. Third, CRP suppresses the proatherogenic effects of macrophages. Fourth, CRP prevents foam cell formation. Fifth, at least in two* in vivo* studies, both human and mouse CRP showed some atheroprotective effects.

## 4. Conclusions

There is no data to suggest that CRP causes a disease. CRP infused in healthy human adults does not result in any significant clinical, hematologic, coagulative, or biochemical changes or any increase in proinflammatory cytokines or acute phase proteins [90]. In case of acute myocardial infarction in model animals, CRP worsens an already existing disease; CRP does what it is programmed to do, that is, to bind to PCh and activate complement, and just in this case CRP does harm. We conclude that CRP is an atheroprotective molecule and, therefore, a host defense protein. CRP mutants (nonnative CRP) capable of efficiently binding to all forms of atherogenic LDL can be evaluated for their effects on the development of atherosclerosis in available animal models to test our conclusion. Administration of exogenously prepared CRP mutant may be useful to capture atherogenic LDL to prevent atherosclerosis. If it turns out that nonnative CRP is indeed atheroprotective, a long-term goal could be to focus on the discovery and design of small-molecule compounds to target CRP (a compound that can change the structure of endogenous CRP) for capturing atherogenic LDL. The purpose of administering a PCh-based compound to target CRP is to inhibit binding of CRP to damaged cells to prevent further damage to myocardial infarcts. As of now, we do not see any need to lower the circulating level of native CRP, as we have suggested previously [91].
